# Direct and indirect effects of marijuana use on the risk of fatal 2-vehicle crash initiation

**DOI:** 10.1186/s40621-020-00276-9

**Published:** 2020-09-14

**Authors:** Stanford Chihuri, Guohua Li

**Affiliations:** 1 Department of Anesthesiology, Columbia University Vagelos College of Physicians and Surgeons, 622 West 168th St, PH5-505, New York, NY 10032 USA; 2 Department of Epidemiology, Columbia University Mailman School of Public Health, 622 West 168th St, PH5-505, New York, NY 10032 USA

**Keywords:** Alcohol, Causal inference, Driving safety, Marijuana, Motor vehicle crashes

## Abstract

**Background:**

Marijuana and alcohol each play a significant role in fatal crash initiation. We decomposed the total effect of marijuana use in the presence or absence of alcohol on fatal crash initiation into direct and indirect effects.

**Methods:**

Pair-matched data on 5856 culpable drivers (initiators) and 5856 nonculpable drivers (noninitiators) involved in the same fatal 2-vehicle crashes recorded in the Fatality Analysis Reporting System between 2011 and 2016 were analyzed using the conditional logistic regression model and the unified mediation and interaction analysis framework.

**Results:**

Crash initiators were more likely than noninitiators to test positive for marijuana (16.1% vs. 9.2%, *P* < 0.001), alcohol (28.6% vs. 9.7%, *P* < 0.001) and both substances (6.3% vs. 1.6%, *P* < .0001). Adjusted odds ratios of fatal 2-vehicle crash initiation revealed a positive interaction on the additive scale between marijuana and alcohol. Of the total effect of marijuana use on fatal 2-vehicle crash initiation, 68.8% was attributable to the direct effect (51.5% to controlled direct effect and 17.3% to reference interaction effect with alcohol) and 31.2% to the indirect effect (7.8% to mediated interaction effect and 23.4% to pure indirect effect through alcohol).

**Conclusion:**

Our results indicate that the increased odds of fatal 2-vehicle crash initiation associated with marijuana use is due mainly to the direct effect.

## Background

Driving under the influence of drugs has increased over the past two decades and poses a serious threat to traffic safety in the United States (Asbridge et al. 2012; Governors Highway Safety Association (GHSA) 2018; Hartman and Huestis 2013). In 2016, 37.9% of fatally injured drivers tested positive for alcohol, 43.6% for nonalcohol drugs, and 50.5% for two or more substances (Governors Highway Safety Association (GHSA) 2018). Marijuana is the most commonly detected nonalcohol drug and its concurrent use with alcohol is the most common polydrug combination among drivers (Berning et al. 2015; Bonar et al. 2018; Governors Highway Safety Association (GHSA) 2018). Although the prevalence of alcohol-impaired driving has declined in recent years in the United States, it still contributes to 28% of all traffic-injury fatalities or 29 deaths daily (National Center for Statistics and Analysis (NCSA) 2017; National Center for Statistics and Analysis (NCSA) 2019a). About one quarter of fatally injured drivers test positive for marijuana (Governors Highway Safety Association (GHSA) 2018). In 2017, 22.1% of adults aged 18 to 25 years reported use of marijuana in the previous month and 11.3% reported driving under the influence of drugs in the past year (Center for Behavioral Health Statistics and Quality (CBHSQ) 2018; Substance Abuse and Mental Health Services Administration (SAMHSA) 2018). Each year, about one million people are arrested for driving under the influence of drugs in the United States (Federal Bureau of Investigations (FBI) 2019). This number is expected to increase as marijuana becomes more permissible and accessible.

As of November 15, 2019, 34 states and the District of Columbia have enacted medical marijuana laws while 11 states and the District of Columbia have legalized recreational marijuana (National Conference of State Legislatures (NCSL) 2019a; National Conference of State Legislatures (NCSL) 2019b). Owing to its putative analgesic effects, state governments are increasingly considering marijuana as a viable alternative to prescription opioids in chronic pain management (Chihuri and Li 2019; National Academies of Sciences, Engineering, and Medicine (NASEM) 2017). For example, in Colorado, New York, and Illinois, individuals with opioid prescriptions or certain health conditions can now legally purchase medical marijuana at a registered dispensary (Quinton 2019). As more states consider legalizing medical and recreational marijuana, it is important to understand the health consequences of marijuana use, such as its effect on driving safety. Currently, 12 states have zero tolerance laws that prohibit driving with any amount of marijuana in the body, 5 states have per se laws that prohibit driving with marijuana in excess of the legal limit, and 1 state has a permissible inference law that permits law enforcement to assume driving under the influence if delta-9-tetrahydrocannabinol (THC) exceeds the allowable threshold (NCSL 2019c). All other states have laws prohibiting driving under the influence of marijuana based on field sobriety tests and observation by law enforcement officers (Wong et al. 2014; Governors Highway Safety Association (GHSA) 2018; NCSL 2019c).

Use of marijuana can slow reaction time, impair judgement and concentration, and decrease psychomotor skills (Downey et al. 2013; Hartman and Huestis 2013; Hartman et al. 2015; Lenne et al. 2010; Lipari et al. 2016; Robbe 1998; Rogeberg and Elvik 2016). Previous epidemiological studies have found a positive interaction on the additive scale between marijuana and alcohol on fatal crash involvement and initiation (Chihuri et al. 2017; Drummer et al. 2004; Dubois et al. 2015; Gjerde et al. 2011; Laumon et al. 2005; Li et al. 2017; Lipari et al. 2016; Rogeberg and Elvik 2016). Experimental studies have also reported additivity at high concentrations of THC and alcohol (Ramaekers et al. 2004; Robbe 1998; Sewell et al. 2009). However, little is known about the causal pathways linking the concurrent use of marijuana and alcohol to increased risks of crash involvement and initiation. Previous studies of polydrug use and driving safety have assessed interaction but not mediation. The traditional approach to mediation analysis is known to have limitations and be susceptible to bias resulting from exposure-mediator interaction (Richiardi et al. 2013). However, recent development in epidemiologic methods has made it possible to simultaneously assess mediation and interaction (Bellavia and Valeri 2018; VanderWeele 2014; Wang and Arah 2015). The counterfactual framework allows for decomposition of the total effect into direct and indirect effects: hence, disentangling the different pathways linking exposure to outcome (Richiardi et al. 2013; VanderWeele 2014). The present study aims to quantify the direct and indirect effects of marijuana use on the risk of fatal 2-vehicle crash initiation through the unified framework for interaction and mediation analysis (VanderWeele 2014). The unified framework allows for further partitioning the direct effect into controlled direct effect and reference interaction effect, and the indirect effect into mediated interaction effect and pure indirect effect.

## Methods

### Data source

Data for this study came from the Fatality Analysis Reporting System (FARS), which is maintained by the National Center for Statistics and Analysis of the National Highway Traffic Safety Board. Since 1975, the FARS has served as the census of fatal motor vehicle crashes occurring on public roads in the United States. Crashes eligible to be recorded in the FARS are those that have resulted in at least one personal fatality (i.e., a driver, passenger, or a non-occupant) within 30 days of the crash (National Highway Traffic Safety Administration (NHTSA) 2018; Wang and Arah 2015). FARS data are collected from various sources, including death certificates, coroner/medical examiner reports, police crash reports, state vehicle registration files, state driver licensing files, emergency medical service reports, and vital statistics (National Center for Statistics and Analysis (NCSA) 2019b; National Highway Traffic Safety Administration (NHTSA) 2018). This study was deemed not human subjects research Under 45 CFR 46 by the Columbia University Intitutional Review Board (New York, NY).

Trained FARS analysts use standardized operational manuals and uniform coding practices to code more than 140 de-identified data elements into as many as 20 data files each year National Center for Statistics and Analysis (NCSA) 2019b). Data files relevant to this study include the accident, vehicle, and person files. The accident file contains environmental and crash circumstances (e.g., road and weather conditions), the vehicle file contains characteristics of the involved vehicles (e.g., make, model, and body type), and the person file includes demographic and other characteristics for each involved person (e.g., driver age, sex, driving history, and drug testing results) National Center for Statistics and Analysis (NCSA) 2019b; National Highway Traffic Safety Administration (NHTSA) 2018). Quality assurance programs automatically check the data for completeness, timeliness, consistency, and accuracy (National Center for Statistics and Analysis (NCSA) 2019b).

In this study, driver-related factors or unsafe driver actions such as lane weaving or speeding (codes 17–60), obtained from police reports and other supporting documents, were used to assign crash responsibility (National Highway Traffic Safety Administration (NHTSA) 2018). For each crash, up to 4 unsafe driver actions were recorded (National Highway Traffic Safety Administration (NHTSA) 2018). Most unsafe driver actions or errors are considered to have contributed to the crash (Blower 1998). In this study, the driver with one or more unsafe driver actions or errors was regarded as the crash initiator, while the other driver without any errors was regarded as the noninitiator. Driving errors are commonly used as a proxy for culpability. Compared to traffic violations that may require legal proof (Blower 1998), driving errors tend to be uniformly applied and to fit the configuration of the crash site, i.e., vehicle positioning, skid marks, and severity of structural damage. Assignment of driving errors is based on the configuration and evidence on the crash scene as well as interviews with witnesses (Blower 1998). Two-vehicle crashes where both drivers made at least one driving error (i.e., shared culpability) were excluded from this study.

### Study design and population

**Fig. 1:**
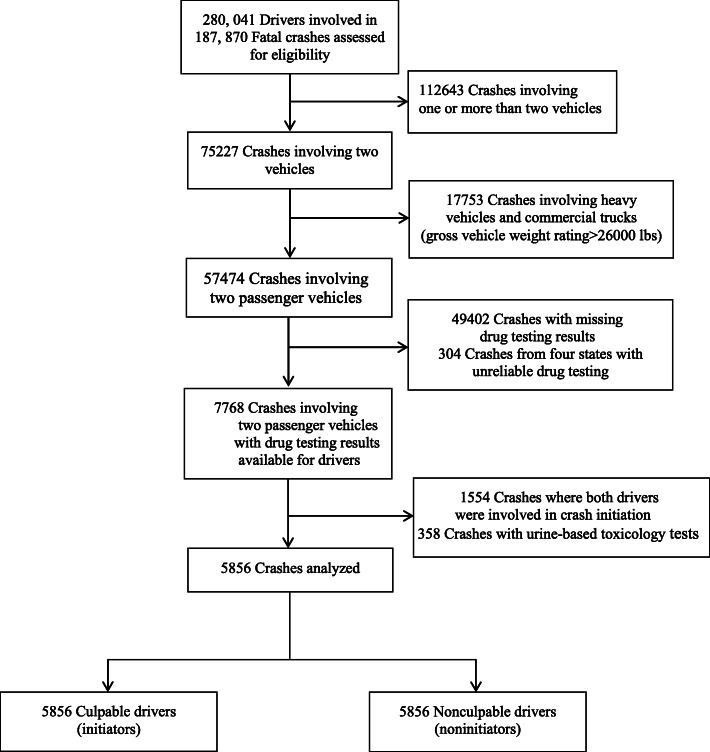
Selection of drivers involved in fatal 2-vehicle crashes, Fatality Analysis Reporting System, 2011–2016

A pair-matched study design was used to assess the individual and joint effects of marijuana and alcohol on the risk of fatal 2-vehicle crash initiation. In this pair-matched study, crash initiators were drivers who were responsible for initiating the fatal 2-vehicle crashes while noninitiators were drivers who were involved in the same 2-vehicle crashes but were not responsible for these crashes. From January 2011 to December 2016, the FARS recorded a total 187,870 fatal crashes involving 280,041 drivers. Excluded from the analysis were 112,643 crashes involving a single vehicle or more than 2 vehicles, 17,753 crashes involving heavy vehicles or commercial vehicles (gross vehicle weight rating 26,000 lbs.), 49,402 crashes with missing toxicological testing results, 304 crashes in Maryland, Montana, New Mexico, and North Carolina with toxicological testing results recorded unreliably in the FARS, 1554 2-vehicle crashes in which both drivers were culpable of crash initiation, and 358 2-vehicle crashes where toxicology tests were based on urine samples (Fig. 1). Included in the study were 5856 pairs of drivers involved in 5856 fatal 2-vehicle crashes with complete toxicological testing data.

### Drug testing assessments

Injury fatalities from motor vehicle crashes are usually investigated by medical examiners or coroners (Executive Office of the President, National Science and Technology Council (EOP-NSTC) 2016). In the United States, 26 states and the District of Columbia have centralized or state medical examiner systems, 12 have coroner systems, and 12 have a county-based systems with a mixture of coroner and medical examiner offices (Centers for Disease Control and Prevention (CDC) 2015; Davis et al. 2015). Overall, approximately 2400 medical-examiner and coroner jurisdictions are responsible for conducting autopsies and performing toxicological tests across the United States (Executive Office of the President, National Science and Technology Council (EOP-NSTC) 2016). For nonfatally injured drivers involved in fatal crashes, blood samples are usually taken at the medical facility where they are treated and those blood samples at admission are routinely used for toxicological analysis (Li et al. 2011).

Toxicological drug tests were conducted on blood or urine specimens using liquid/gas chromatography and radioimmunoassay techniques for screening, and liquid/gas chromatography and mass spectrometry for confirmation (Kaplan et al. 2006; Li et al. 2011). All drivers included in this study had at least one toxicological drug test based on a blood specimen. Prior to 2018, the FARS recorded up to 3 nonalcohol drugs. In instances where a drug metabolite was detected, only the parent drug was recorded National Center for Statistics and Analysis (NCSA) 2019b; National Highway Traffic Safety Administration (NHTSA) 2018). If more than 3 nonalcohol drugs were detected, the FARS recorded the drugs in the following priority: narcotics, depressants, stimulants, marijuana, and other drugs (Kaplan et al. 2006; National Highway Traffic Safety Administration (NHTSA) 2018). In the present study, marijuana refers to cannabinoids such as THC and/or other cannabinoid metabolites (codes 600–695) (National Center for Statistics and Analysis (NCSA) 2019b). Blood alcohol concentrations (BACs) were measured and recorded separately from nonalcohol drugs and a BAC of 0.01 g/dL or greater was considered alcohol-positive (National Center for Statistics and Analysis (NCSA) 2019b; National Highway Traffic Safety Administration (NHTSA) 2018). We also analyzed BAC data as a 3-level categorical variable (BACs < 0.01, 0.01–0.07, and ≥ 0.08 g/dL).

### Statistical analysis

Frequency distributions of driver characteristics were tabulated by crash initiation status. The McNemar’s test was used to compare initiators and noninitiators on driver characteristics such as age, sex, marijuana testing result, alcohol testing result, driving history within the previous 3 years, and survival status. The Pearson χ^2^ test was use to compare initiators and noninitiators on age categories and BAC levels. The Cochran Armitage trend test was used to assess the changes in the prevalence of marijuana detected in drivers over the study period. Conditional logistic regression modeling was used to estimate crude and adjusted odds ratios (ORs) and 95% confidence intervals (CIs) of crash initiation associated with marijuana use, alcohol use, and other driver characteristics. To assess separate and joint effects of marijuana and alcohol, drivers testing negative for marijuana and alcohol were assigned as the reference group. The interaction of marijuana and alcohol was assessed on the multiplicative and additive scales. Additive interaction was assessed using 3 statistics: the relative excess risk due to interaction (RERI), attributable proportion due to interaction (AP), and the synergy index (S). The corresponding 95% CIs were computed using a method suggested by Zou (2008).

**Fig. 2:**
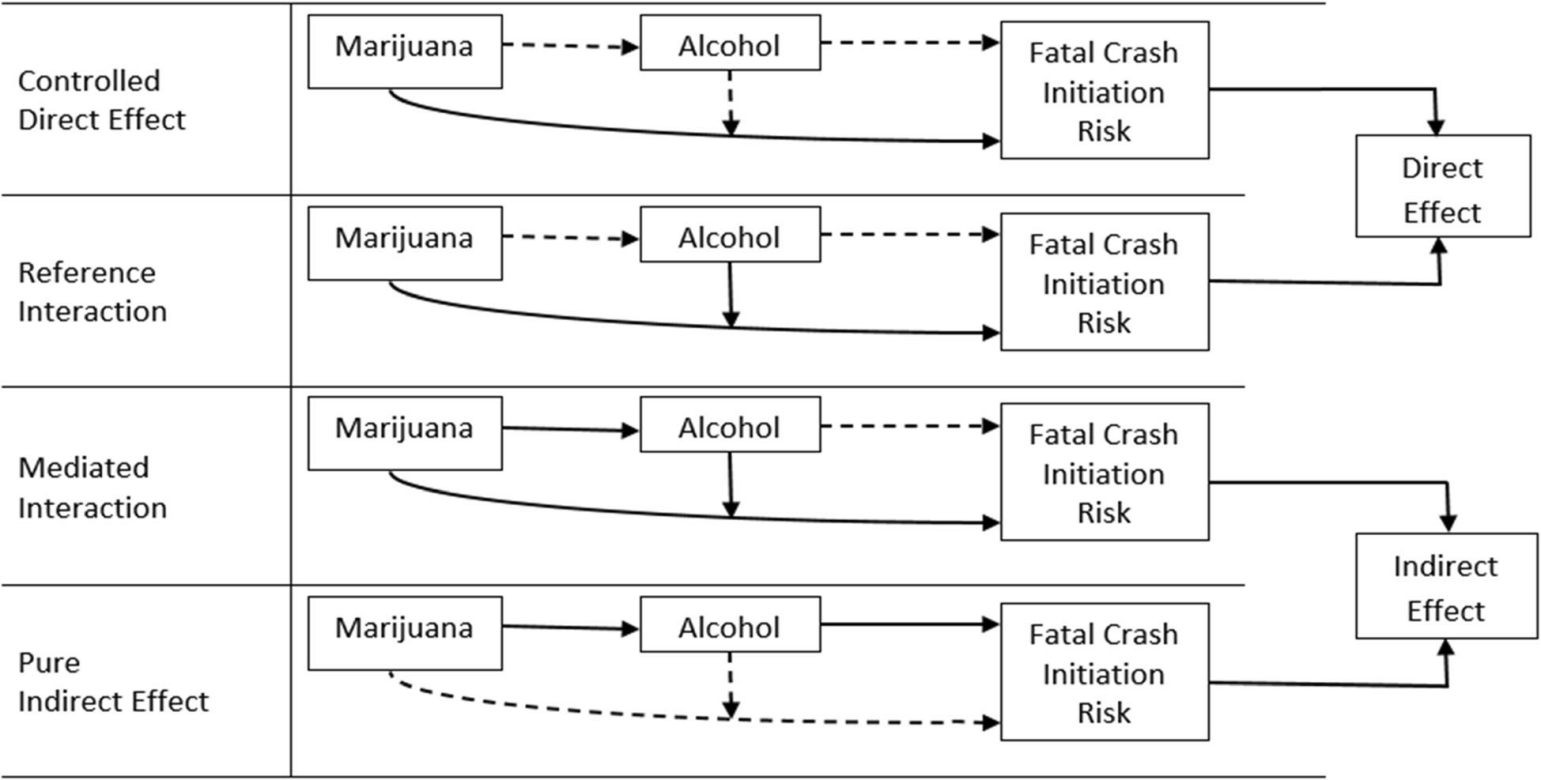
Graphical explanation of the four component effects of marijuana use on fatal 2-vehicle crash initiation in the presence of alcohol under the unified framework for mediation and interaction analysis proposed by WanderWeele (2014)

The unified framework for interaction and mediation analysis developed by VanderWeele (2014) was used to quantify the direct and indirect effects of marijuana use on the risk of crash initiation (Fig. 2). The total effect of marijuana was decomposed into 4 components: 1) controlled direct effect, which refers to the effect of marijuana on the risk of crash initiation in the absence of alcohol (i.e., the portion of the total effect of marijuana that is due to neither interaction nor mediation); 2) reference interaction, which refers to the combined effect of marijuana and alcohol on the risk of crash initiation if alcohol is not in itself necessary for crash initiation from marijuana use (i.e., the portion of the total effect of marijuana that is due to interaction only); 3) mediated interaction, which refers to the combined effect of marijuana and alcohol on the risk of crash initiation if alcohol is necessary for crash initiation from marijuana use (i.e., the portion of the total effect of marijuana that is due to both mediation and interaction); and 4) pure indirect effect, which refers to the effect of marijuana on the risk of crash initiation operated through alcohol as the mediator (i.e., the portion of the total effect of marijuana that is due to mediation through alcohol only). The direct effect comprises the controlled direct effect and reference interaction whereas the indirect effect is made up of the mediated interaction and pure indirect effect (Fig. 2). All data analyses were performed using SAS, version 9.4 (SAS Institute Inc., Cary, NC). Statistical significance was set at *P* < 0.05 for 2-tailed tests.

## Results

Compared with drivers excluded from the study due to missing or incomplete drug testing results, those included in the analysis were slightly younger (mean age: 42.2 years, standard deviation:18.3 years vs. mean age: 43.7 years, standard deviation: 19.1 years, *P* < 0.0001), more likely to be male (71.3% vs. 70.3%, *P* = 0.028), and more likely to be involved in a crash in the previous 3 years (23.1% vs. 22.2%, *P* = 0.026). Drivers included in the analysis did not significantly differ from the excluded drivers with regards to survival status and other driving histories in the previous 3 years, such as license suspension, driving-while-intoxicated conviction, and speeding conviction.

The most common driving error leading to fatal 2-vehicle crashes was failure to keep in proper lane (24.8%), followed by failure to yield right of way (18.9%) (Table 1). Of the driving errors committed by drivers testing positive for marijuana, 22.6% were failure to keep in proper lane and 22.4% by speeding (Table 1).

**Table 1:** Frequency distribution of driving errors involved in 5856 fatal 2-vehicle crashes by marijuana positivity status, Fatality Analysis Reporting System, 2011–2016

Type of driving error	Positive for marijuana No. (%)	Negative for marijuana No. (%)	Total No. (%)
Failure to keep in proper lane	322 (22.6)	1577 (25.3)	1899 (24.8)
Failure to yield right of way	148 (10.4)	1299 (20.8)	1447 (18.9)
Driving too fast for conditions or in excess of posted maximum	320 (22.4)	1037 (16.6)	1357 (17.7)
Failure to obey actual traffic signs, traffic control devices, or traffic officers	150 (10.5)	684 (11.0)	834 (10.9)
Operating the vehicle in an erratic, reckless, careless, or negligent manner or at erratic or suddenly changing speeds	110 (7.7)	339 (5.4)	449 (5.9)
Driving on wrong side of the road	107 (7.5)	541 (8.7)	648 (8.4)
Manslaughter or homicide or other assault	93 (6.5)	313 (5.0)	406 (5.3)
Making an improper turn	24 (1.7)	132 (2.1)	156 (2.0)
Passing with insufficient distance or inadequate visibility or failing to yield to overtaking vehicle	31 (2.2)	94 (1.5)	125 (1.6)
Passing where prohibited	60 (4.2)	16 (0.3)	76 (1.0)
Any other	62 (4.3)	211 (3.4)	273 (3.6)
Total^a^	1427 (100.0)	6243 (100.0)	7670 (100.0)

^a^Total exceeds the number of crashes because more than 1 error could be recorded in each crash

Between 2011 and 2016, the prevalence of marijuana increased from 14.0 to 18.1% (*P* = 0.0001) among crash initiators, and from 7.0 to 13.4% (*P* < 0.0001) among noninitiators. Overall, crash initiators were more likely than noninitiators to test positive for marijuana (16.1% vs. 9.2%, *P* < 0.001), alcohol (28.6% vs. 9.7%, *P* < 0.001), and both substances (6.3% vs. 1.6%, *P* < 0.0001). Initiators were more likely than noninitiators to be under 35 years of age (50.8% vs. 33.8%, *P* < 0.0001), to have died in the crash (55.5%vs. 41.9%, *P* < 0.0001), and to have had a crash (23.0% vs. 19.2%, *P* < 0.0001), a driving-while-intoxicated conviction (5.8% vs. 2.8%, *P* = 0.0009), a speeding conviction (21.4% vs. 17.1%, *P* < 0.0001), or license suspension (18.7% vs. 10.0%, *P* < 0.0001) within the previous 3 years (Table 2).

**Table 2:** Characteristics of drivers involved in fatal 2-vehicle crashes by crash initiation status, Fatality Analysis Reporting System, 2011–2016

Driver characteristic	Initiators^a^ *n* = 5856 N (%)	Noninitiators^b^ *n* = 5856 N (%)	*P* value
Age, years			
16–24	1517 (25.9)	899 (15.4)	< 0.0001
25–34	1457 (24.9)	1078 (18.4)	
35–44	843 (14.4)	981 (16.8)	
45–54	713 (12.2)	1085 (18.6)	
55–64	549 (9.4)	985 (16.8)	
≥ 65	775 (13.2)	822 (14.1)	
Sex			
Female	1679 (28.7)	1723 (29.4)	0.4268
Male	4175 (71.3)	4133 (70.6)	
Crash in the past 3 years			
Yes	1309 (23.0)	1098 (19.2)	< 0.0001
No	4385 (77.0)	4606 (80.8)	
DWI conviction in the past 3 years			
Yes	338 (5.8)	163 (2.8)	0.0009
No	5494 (94.2)	5677 (97.2)	
Speeding conviction in the past 3 years			
Yes	1250 (21.4)	1000 (17.1)	< 0.0001
No	4582 (78.6)	4840 (82.9)	
License suspension in the past 3 years			
Yes	1090 (18.7)	582 (10.0)	< 0.0001
No	4742 (81.3)	5258 (90.0)	
Tested positive for marijuana			
Yes	942 (16.1)	541 (9.2)	< 0.0001
No	4914 (83.9)	5315 (90.8)	
Tested positive for alcohol			
≥ 0.01	1673 (28.6)	569 (9.7)	< 0.0001
0.00	4183 (71.4)	5287 (90.3)	
Survival status			
Dead	3253 (55.5)	2456 (41.9)	< 0.0001
Alive	2603 (44.5)	3400 (58.1)	

*Abbreviation*: *DWI* Driving while intoxicated

^a^Among initiators 2 had missing data on age, 2 on sex, 24 on speeding conviction, 24 on license suspension, 24 on DWI conviction, and 162 on crash within the past 3 years

^b^Among noninitiators, 2 had missing data on age, 16 on speeding conviction, 16 on license suspension, 16 on DWI conviction, and 152 on crash within the past 3 years

**Fig. 3:**
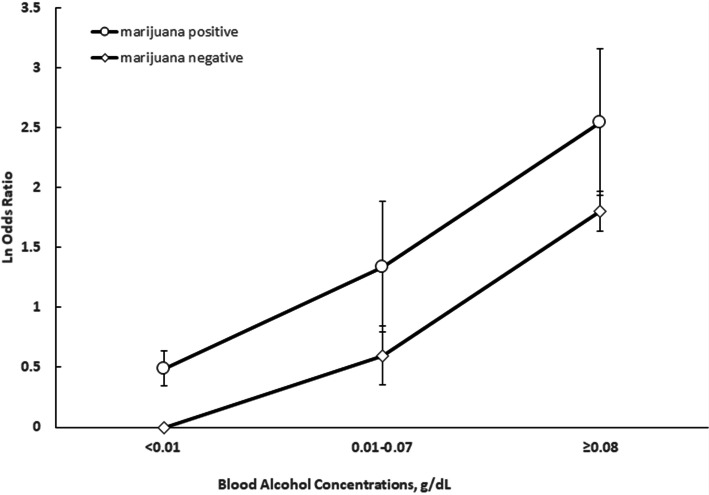
Estimated adjusted odds ratios (ORs) and 95% confidence intervals (95% CIs) of fatal 2-vehicle crash initiation according to marijuana positivity status and blood alcohol concentrations, Fatality Analysis Reporting System, 2011–2016

Marijuana use and alcohol use were each associated with a significantly increased risk of fatal 2-vehicle crash initiation when adjusting for driver age, sex, and driving history within the previous 3 years (Table 3). Compared to drivers who tested negative for both alcohol and marijuana, the estimated odds of fatal crash initiation increased 1.5-fold for those testing positive for marijuana and negative for alcohol, 5-fold for those testing negative for marijuana and positive for alcohol, and 6.8-fold for those testing positive for both marijuana and marijuana (Table 3). The odds of crash initiation increased with BACs regardless of marijuana positivity status (Fig. 3).

**Table 3:** Estimated odds ratios (ORs) and 95% confidence intervals (CIs) of fatal 2-vehicle crash initiation by driver marijuana and alcohol positivity status, Fatality Analysis Reporting System, 2011–2016

Marijuana	Alcohol	Crude		Adjusted^a^	
		OR	95% CI	OR	95% CI
Negative	Negative	1.00	reference	1.00	reference
Positive	Negative	1.81	1.58, 2.09	1.53	1.31,1.77
Negative	Positive	4.70	4.11, 5.39	4.95	4.28, 5.72
Positive	Positive	7.46	5.72, 9.72	6.76	5.12, 8.94

*Abbreviations*: *OR* Odds ratio, *CI* Confidence interval

^a^Adjusted for age, sex, and previous driving history within the past 3 years (i.e., crash, license suspension, driving while impaired conviction, and speeding conviction)

There was no significant interaction on the multiplicative scale as shown by the interaction term of marijuana and alcohol (β = − 0.1103, *P* = 0.4982). However, there was a significant interaction on the additive scale as assessed by all 3 statistics: RERI = 1.29 (95% CI: 0.40, 3.44), AP = 0.19 (95% CI: 0.09, 0.38), and S = 1.29 (95% CI: 1.02, 1.81). Of the total effect of marijuana on fatal crash initiation risk, 68.8% was attributed to direct effect (51.5% to controlled direct effect and 17.3% to reference interaction with alcohol) and 31.2% to indirect effect [7.8% to mediated interaction through alcohol and 23.4% to pure indirect effect (Table 4)].

**Table 4:** Four-way decomposition of the total effect of marijuana use on fatal 2-vehicle crash initiation in the presence of alcohol

Component	Log (Odds Ratio)	95% Confidence Interval	Effect Proportion	*P* value
**Total Effect**	**0.499**	**0.31, 0.66**	**1.00**	**< 0.001**
Controlled Direct Effect	0.257	0.16, 0.36	0.515	< 0.001
Reference Interaction	0.086	−0.04, 0.21	0.173	0.181
Mediated Interaction	0.039	−0.02,0.10	0.078	0.205
Pure Indirect Effect	0.117	0.05, 0.18	0.234	0.001

## Discussion

Results of this study indicate that marijuana use and alcohol use are each associated with a significantly increased risk of fatal 2-vehicle crash initiation and that concurrent use of marijuana and alcohol confers a significant interaction effect on the risk of fatal crash initiation on the additive scale. Furthermore, over two-thirds of the total effect of marijuana use on fatal crash initiation are attributed to the direct effect, including 52% due to controlled direct effect and 17% due to reference interaction. Our findings shed more light on the causal role of marijuana use in crash initiation. Specifically, the decomposition analysis indicates that the increased risk of crash initiation associated with marijuana use is operationalized through dual pathways, with 69% being attributed to the direct effect and 31% to the indirect effect. These results are generally consistent with findings from experimental studies (Crancer Jr et al. 1969; Moskowitz et al. 1976; Ramaekers 2003; Smiley et al. 1981) and epidemiologic studies (Asbridge et al. 2012; Li et al. 2012; Rogeberg and Elvik 2016).

There is a paucity of research assessing the mediation effect of marijuana by alcohol on crash risk and crash initiation. Our study reveals that about 23% of the total effect of marijuana use on fatal 2-vehicle initiation is due to mediation through alcohol, which is consistent with the additive effect model of risk factors where one exposure contributes to another exposure that cumulatively increase the risk of the outcome (Bean et al. 2019). Since the risk of crash initiation increases with BACs, the magnitude of the mediation effect of marijuana by alcohol is likely to increase with BACs in a dose-response fashion. Results of the 4-way decomposition also show that 25% of the total effect was attributed to interaction (i.e., mediated interaction and reference interaction). Although the individual components of mediated interaction and reference interaction are not statistically significant, the overall interaction effect on the additive scale is statistically significant.

Assessing interactions between alcohol and other drugs on driving safety may help identify subgroups of drivers to maximize public health impact in resource allocation and risk reduction (Blot and Day 1979; Luedtke and Van der Laan 2016; Rothman et al. 1980, 2008; Saracci 1980; VanderWeele 2015). However, the relative effects across subgroups may change depending on the scale (i.e., multiplicative vs. additive). The “interaction continuum” proposed by VanderWeele (2019) provides an ordinal metric for gauging the strength of interaction across scales, from the strongest (positive-multiplicative positive-additive) to the weakest (inverted interaction) (VanderWeele 2019). Results from this study suggest that concurrent use of marijuana and alcohol confers an effect on fatal crash initiation that corresponds to the second strongest form of interaction on the interaction continuum, namely no-multiplicative positive additive (VanderWeele 2019).

According to VanderWeele (2014), the unified framework for mediation and interaction analysis is based on four assumptions: 1) the effect of marijuana use on crash initiation is unconfounded conditional on baseline covariates; 2) the effect of alcohol use on crash initiation is unconfounded conditional on baseline covariates and marijuana use; 3) the effect of marijuana use on alcohol use is unconfounded conditional on covariates; and 4) none of the confounders of alcohol use on crash initiation are affected by marijuana use. Although it is difficult to rigorously evaluate each of the assumptions, our study takes into consideration these assumptions through design and analytical approaches. First, the pair-matched design ensures that crash initiators and noninitiators are matched on weather, road and traffic conditions, location and time of the crash, regulation, toxicological testing protocol, and other spatiotemporal and environmental factors. Second, the conditional logistic regression model controls for driver age, sex, and driving history in the previous 3 years (i.e., speeding convictions, DWI, license suspension, and crashes). Finally, assignment of crash initiation status was based on driving errors because they do not require legal proof and they tend to be uniformly applied (Blower 1998) and have been widely used in previous culpability studies.

Our study shows that the prevalence of marijuana detected in drivers involved in fatal 2-vehicle crashes has increased steadily in the past decade. This is likely due in a large part to the increased permissibility and availability as more states have enacted laws to legalize marijuana for medical and recreational use. Marijuana use is associated with impairment of psychomotor skills necessary to operate a motor vehicle safely such as reaction time which may lead to failure to yield right of way (Chihuri et al. 2017; Downey et al. 2013; Hartman and Huestis 2013; Lenne et al. 2010; Lipari et al. 2016; Robbe 1998; Rogeberg and Elvik 2016). Marijuana use may also impair higher-level driving skills such as hazard perception, risk management and self-control, which may lead to failure in lane tracking and other driving errors (Downey et al. 2013; Hartman and Huestis 2013; Lenne et al. 2010; Lipari et al. 2016; Robbe 1998; Rogeberg and Elvik 2016). In the present study, failure to keep in proper lane and failure to yield right of way were the two most frequently identified driving errors leading to fatal 2-vehicle crashes. Although alcohol use remains a much stronger risk factor for crash initiation, marijuana is associated with elevated risk both in the presence or absence of alcohol through direct and indirect pathways. As such, policymakers should consider developing countermeasures that target use of specific substances as well as polysubstance use, such as concurrent use of alcohol and marijuana and THC-infused alcoholic beverages.

This study had several limitations. First, testing positive for marijuana indicates marijuana use but does not necessarily imply marijuana-induced impairment at the time of crash. Given that marijuana metabolites stay longer in the urine compared to blood (AIT Laboratories 2011), we restricted the analysis to toxicological tests based on blood specimens only. Second, drug testing data are available for only about 40% of drivers involved in fatal crashes and drug testing and recording procedures may differ across states and jurisdictions (Berning and Smither 2014). Drivers with missing or incomplete toxicological testing data were excluded from our analysis. Although this data limitation may pose a threat to the external generalizability of our findings, the study design should help ensure a reasonably high level of internal validity of the results as initiators and noninitiators were paired-matched on weather, road condition, location, time of crash, traffic regulation, toxicological testing protocol, and other tempo-spatial factors. It is noteworthy that, despite the control for tempo-spatial factors through pair-matching, the study results may still be susceptible to biases from unmeasured confounders on the individual level, such as socioeconomic status, chronic substance use behavior, and comorbidities. Finally, the FARS does not record THC concentrations for drivers who tested positive for marijuana and thus we are unable to assess the dose-response effect of marijuana on crash initiation.

## Conclusions

Results of this study indicate that marijuana use and alcohol use are each associated with a significantly increased odds of fatal 2-vehicle crash initiation. When used together, marijuana and alcohol confer a positive additive interaction effect on the odds of fatal 2-vehicle crash initiation. The decomposition analysis shows that over two-thirds of the total effect of marijuana on crash initiation are due to the direct effect whereas the remaining is due to the indirect effect through alcohol. Given the increasing prevalence of marijuana use and concurrent use of marijuana and alcohol in the driver population, multifaceted intervention programs are needed to target driving under the influence of specific substances and driving under the influence of polysubstances.

## Data Availability

Data from this study came from the Fatality Analysis Reporting System (FARS), maintained by the National Highway Traffic Safety Administration (NHTSA). These data are publically available and may be downloaded from https://www.nhtsa.gov/research-data/fatality-analysis-reporting-system-fars.

## Abbreviations

AP: Attributable proportion due to interaction
BAC: Blood alcohol concentration
CI: Confidence interval
DWI: Driving while intoxicated
FARS: Fatality Analysis Reporting System
OR: Odds ratio
RERI: Relative excess risk due to interaction
S: The synergy index
THC: Delta-9-tetrahydrocannabinol
